# Autologous micrografts and methotrexate in plantar erosive lichen planus: healing and pain control. A case report

**DOI:** 10.1080/23320885.2020.1848434

**Published:** 2020-12-28

**Authors:** G. Miotti, N. Zingaretti, G. F. Guarneri, V. Manfrè, E. Errichetti, G. Stinco, P. C. Parodi

**Affiliations:** a Department of Plastic and Recostructive Surgery, “Santa Maria della Misericordia” University Hospital, Udine, Italy;; b Institute of Dermatology, Department of Medicine, “Santa Maria della Misericordia” University Hospital, Udine, Italy

**Keywords:** ELP;, *Rigenera* micrografts;, pain treatment;, wound healing;, regenerative surgery

## Abstract

Erosive lichen planus is an uncommon variant of lichen planus. We report a case of longstanding and refractory plantar ELPs causing disabling and opiate-resistant pain treated with ‘classic’ meshed skin graft combined with *Rigenera^®^* micrografts. After approximately 9 months follow-up, no clinical recurrence or pain were observed. Erosive lichen planus (ELP) is an uncommon variant of lichen planus, involving oral cavity and genitalia and, less often plantar areas, where it usually presents with chronic erosions of the soles, along with intense, disabling pain and progressive loss of toenails. An abnormal immune cellular response (CD8+ lymphocytes and macrophages) and the consequent altered production of multiple mediators (interleukin-12, interferon-γ, tumor necrosis factor-α, RANTES and MMP-9), seem to play a crucial role in the pathogenesis, although the etiology remains uncertain. From a histological point of view, ELP shows keratinocyte apoptosis, intense inflammatory response and basal epithelial keratinocytes TNF-α overexpression. Several therapies have been proposed, with variable and controversial results. While topical corticosteroids and topical calcineurin inhibitors are the treatments of choice for localized forms, short pulses of systemic glucocorticoids, phototherapy, and systemic immunosuppressants are recommended for generalized cases. Surgery has been reported as a possible therapeutic option in refractory and stable cases with localized lesions, either alone or with cyclosporine. Herein, we report a case of longstanding and refractory plantar ELPS causing disabling and opiate-resistant pain treated with ‘classic’ meshed skin graft combined with *Rigenera®* micrografts.

## Case report

**Figure 1.**
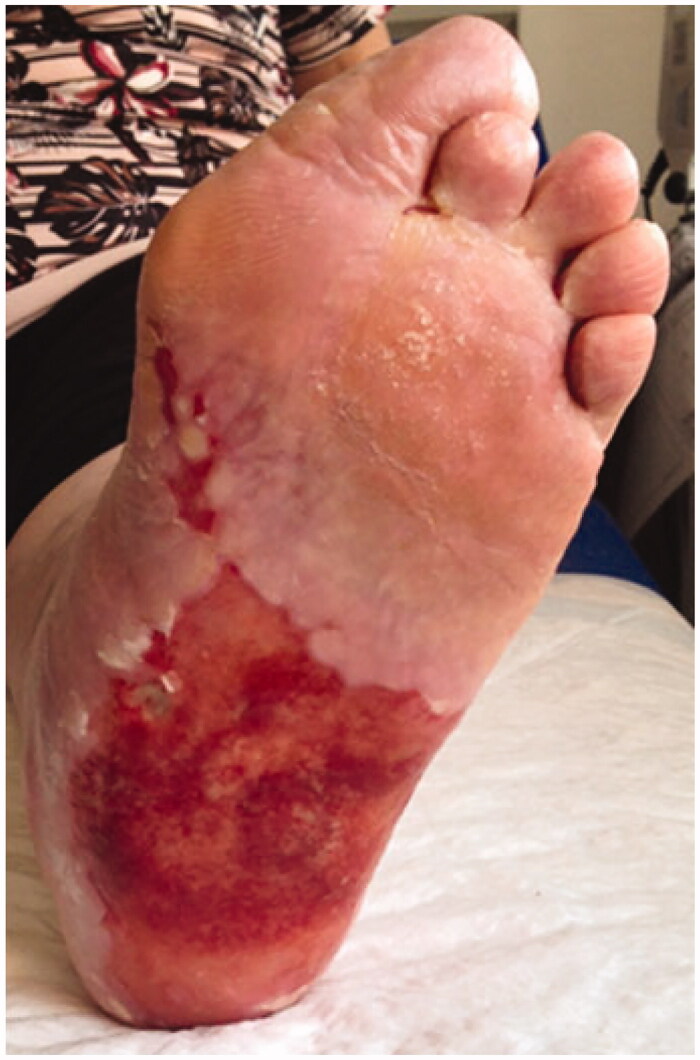
Extensive, malodorous and irregular ulcer, having well-defined margins and a reddish and friable base.

A 65-year-old Caucasian female presented with a 6-year history of a wide and extremely painful ulceration on the sole of her left foot, histologically diagnosed as erosive lichen planus. Over the years she had undergone several topical and systemic treatments, including steroids, antibiotics, retinoid and mycophenolate mophetil, with transient and incomplete results.Skin examination revealed an extensive, malodorous and irregular ulcer, having well-defined margins and a reddish and friable base (Figure 1).

Patient reported strong pain and a burning sensation which impeded her to walk. Absence of toenails was also evident. Medical history included many comorbidities, viz. type-2 diabetes, hypertension, dislipidemia, obesity, chronic renal failure, IgG k gammopathy, multifactorial anemia, erosive gastritis, and esophagitis. Laboratory tests showed only some alterations related to the patient's comorbidity and an increase in the Erythrocyte Sedimentation Rate (38 mm/h) and the C-reactive protein level (1.56 mg/L). Anti-hepatitis C and B viruses’ antibodies were negative.

Steroid topical preparations were applied under occlusive wet dressings and systemic Methotrexate was started with a dosage of 15 mg once a week. The ulcer showed a moderate improvement with increase of granulation tissue at the base but without effect on the pain (steadily with NRS scale score >7), which turned out to be intense and resistant to any type of analgesic, including opiates, with consequent compromising of her life quality. For this reason, we proposed her a surgical treatment, consisting in the combination of a meshed split thickness autologous skin graft, and autologous skin micro-grafts (obtained by *Rigenera^®^*).

**Figure 2.**
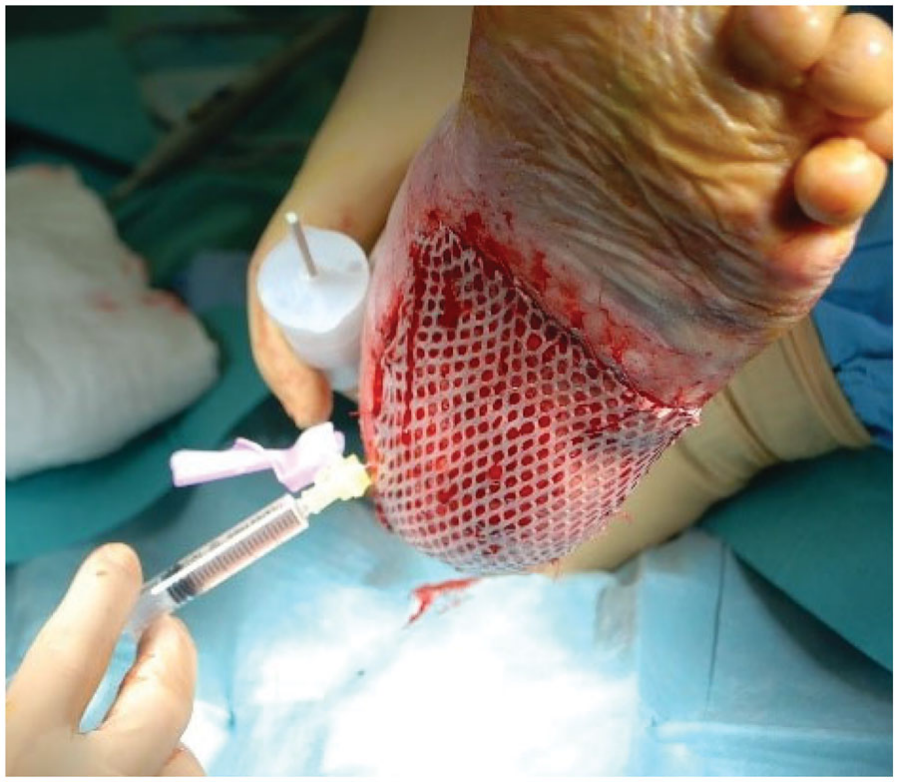
Injection of solution containing autologous micrografts obtained with *Rigenera*® protocol.

Surgery was performed under local anesthesia with sedation. After surgical debridement of the plantar lesion, two 3 mm^2^ skin punch biopsies where taken from the left thigh and processed through *Rigenera^®^* at 80 rpm for 2 min in 3 ml of 0.9% NaCl saline solution, each. The resulting solution was injected on margins and bottom of the lesion (Figure 2). Then, the area was covered with a 1:2 meshed split thickness skin graft taken from her left thigh, and a nonadherent gauze and sterile gauze compression dressing.

**Figure 3.**
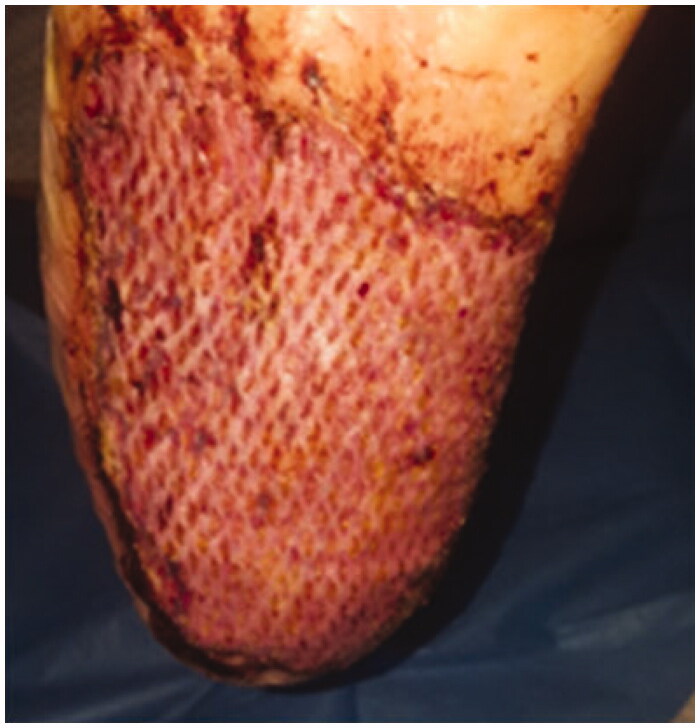
Skin engraftment after 5 days.

**Figure 4.**
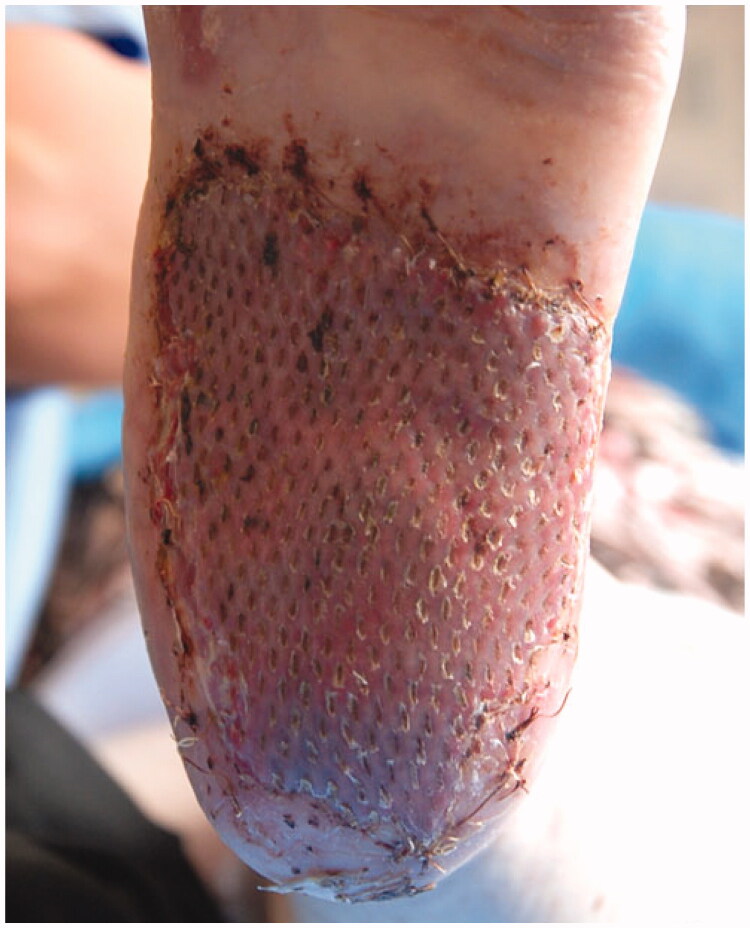
Skin engraftment after 14 days.

After 5 days, the compression dressing was removed, showing a gook skin engraftment, and complete re-epithelialization after 14 days (Figures 3 and 4).

The patient referred immediate, complete and stable regression of pain and local symptoms.

After hospital discharge, weekly medications in outpatient service followed, with progressive improvement of the skin engraftment and no reappearance of pain. Methotrexate was continued with a reduced dose of 10 mg once a week. The patient has been followed-up for approximately 9 months and neither clinical recurrence nor pain occurred.

## Discussion

The repair and regeneration of human tissues is particularly difficult and given that regenerative ability declines with increasing age. The dramatic rise in the ageing population worldwide determined an increasing need for innovative approaches. Many approaches can be used when the normal process of tissue regeneration is impaired or simply insufficient, such as grafting which includes autografts, allografts, xenografts, and biomaterial substitutes [1,2].

Treatment of ELP is notoriously difficult, because of its frequent resistance to topical or systemic therapies and high rates of recurrence. Multiple treatment options have been proposed, but none of them has consistently demonstrated satisfactory remission of symptoms. Surgery could be an important alternative treatment for this disease [3].

In a previous case of ELP, the patient demonstrated a notable clinical response using cyclosporine and skin grafting [4]. In the present patient, the numerous comorbidities prevented the use of different immunosuppressants. As prescribed by Dermatologists, methotrexate was here used, albeit in the literature there was no previous data on its use in plantar ELP. Notably, even though we did not observe complete disease resolution, we obtained partial reduction of inflammation and increase of granulation tissue, that might have favored the surgical approach.

At the same time, it is known that immunosuppressive therapy causes an increased risk of delayed wound healing, infections and failure of the skin grafts engraftment [5,6]. Referring to all these variables, we had to consider an alternative solution, which would accelerate reepithelization, such as using micro-grafts. In our experience we have already tested their validity in other wound types [7].

We choose autologous skin micro-grafts obtained by *Rigenera^®^* technology using a class I medical device called Rigeneracons (Human Brain Wave, Italy), already used in various fields of medicine and dentistry [8–10].

We applied the same *Rigenera^®^* protocol reported in the literature [8–13].

**Figure 5.**
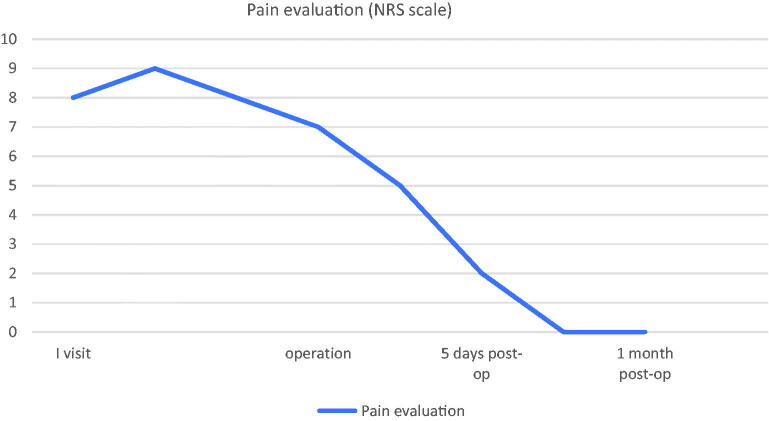
Pain evaluation using NRS scale.

Micrograft technique in this patient had five functions:

1. Acceleration of skin wound healing: studies have shown a more rapid reepithelization when micro-grafts are associated with classic skin graft, with a better result also in terms of skin quality [11,12]. *In vitro* results show that the micrografts obtained with *Rigenera^®^* maintain a high cell viability, despite the disaggregation needed to obtain them. In particular, micrografts were described rich in mesenchymal stem cells (MSCs), with high CD90, CD73, and CD105 positivity [13,14]. Preclinical and clinical trials show that MSC therapy accelerates wound closure suggesting that it can be promising for treating wounds with delayed healing [15,16]. In addition, MSCs promote different stages of the wound repair process through modulation of inflammation, promotion of angiogenesis, and stimulation of cell movement during epithelial remodeling [17]. On the basis of these evidences, we can suppose that the regenerative properties of micrografts can be in part determined by MSCs activity. This supports the capacity of these micro-grafts to improve wound healing [18,19], and having a more fast and complete reepithelialization respect to the other advanced dressings, with excellent results in terms of clinical efficacy and demonstration of the regenerative capacity of this method [7].
2. Regenerative effect: in micrografts, the keratinocytes and fibroblasts are surrounded by a supportive microenvironment, increasing the regenerative capacity of the cells when transplanted [12]. The goal of regeneration is implanting so small cells to ensure an optimal grafting, versus the critical points of a conventional autologous grafts: their limited regenerative capacity and the percentage of cellular death in the receiving site [20]. Cell characterization by flow cytometry analysis shows a heterogeneous pool of cells [18,19]. The specific cell population of these micrografts includes progenitor cells [19], which in association with the fragment of the Extracellular Matrix (ECM) and growth factors derived by starting tissue, initiate biological processes of cell proliferation and differentiation enhancing the wound healing process [7,11].
3. Anti-inflammatory effect: this micrograft technique allows to select factors such as TGF-β_1_ (which acts both as a promoter of tissue repair and as a suppressor of excessive inflammation) [18,21,22,23,24] and alpha-smooth muscle actin (α-SMA), which plays a central role in the healing process (wound contraction) and which is produced from myofibroblasts, once they have differentiated from fibroblasts upon stimulation of TGF-β_1_ present in the ECM [11].
4. Foot sole sensitivity: TGF-β_1_ appears to stimulate the de-differentiation of glial cells to neural crest stem cells. These cells, in turn, appear to be involved in the progression of healing and the restoration of peripheral sensitivity [22].
5. Pain reduction: our patient developed immediate and permanent pain reduction (NRS 2 at first control at 5 days, 0 at 14 days, Figure 5). Although in literature the reduction of pain after treatment with micrograft has already been reported [6], this result is to be ascribed to the aforementioned mediators obtained with this method [11,12]

The impressive clinical outcomes combined with the laboratory tests, demonstrate that this technology is really able to stimulate skin regeneration. On the basis of this evidence, *Rigenera®* can offer an efficient and promising strategy to improve symptoms and wound healing in patients who exhibit painful and nonhealing wounds.

## Conclusion

In this case report, we presented an alternative use of autologous micro-grafts obtained with the *Rigenera^®^* protocol, i.e. treatment of chronic inflammatory condition as ELP. We believe that the addition of autologous micrografts to the meshed split thickness skin grafts not only promoted the skin engraftment and the healing of the erosive lesions, but also helped to reverse almost instantly the pain, thanks to the immunosuppressive and anti-inflammatory properties.

Finally, as showed in our instance, methotrexate could have a role in preparing the lesions to the surgical procedure and in ensuring prolonged post-surgery remission. Yet, future studies are needed to confirm such a speculation as we did not have data in the post-suspension period because we did not withdraw the medication to see the disease evolution due to ethical reason.

Declarations of interest: All authors certify that they have NO affiliations with or involvement in any organization or entity with any financial interest (such as honoraria; educational grants; participation in speakers’bureaus; membership, employment, consultancies, stock ownership, or other equity interest; and expert testimony or patent-licensing arragements), or non-financial interest (such as personal or professional relantionship, allilations, knowledge or beliefs) in the subject matter or materials discussed in this manuscript.
